# Supplementary material for: Single-molecule electrical contacts on silicon electrodes under ambient conditions
Source: Nat Commun. 2017 Apr 13;8:15056. doi: 10.1038/ncomms15056 (PMC5399279; doi:10.1038/ncomms15056)
Supplement: Supplementary Information — Supplementary Figures, Supplementary Table, Supplementary Notes, Supplementary Methods and Supplementary References [file ncomms15056-s1.pdf]

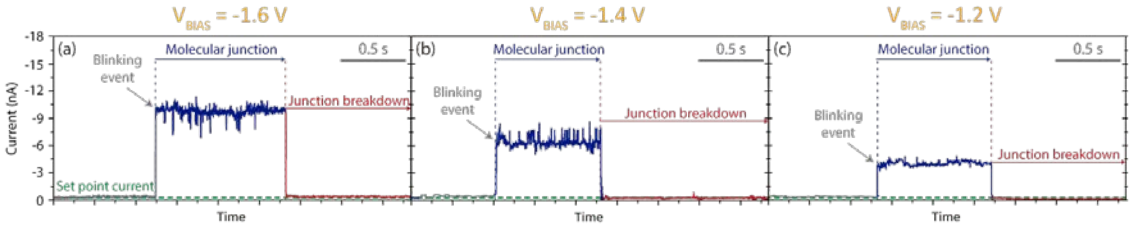

***Supplementary Figure 1* | Blinking experiments of Au-Si junctions.** Representative individual blinking current traces of 1,8 nonadiyne molecules on Si_{HD} under a surface bias of -1.6 V (a), -1.4 V(b) and -1.2 V(c). See *Supplementary Note 1* for detailed information.

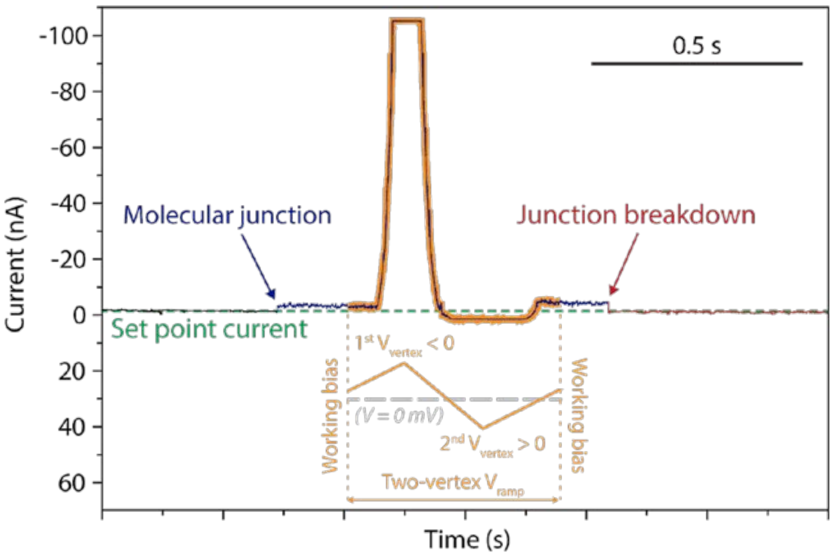

***Supplementary Figure 2* | Blinking of Au-(1,8-nonadiyne)-Si_{LD} and Au-gap-Si junctions.** Current response during the applied "2-vertex voltage ramp" of a representative 1,8-nonadiyne single-molecule junction formed between an Au tip and the low doped n-type silicon substrate electrodes showing a current increase in the forward bias while limited current in the reverse bias is observed.

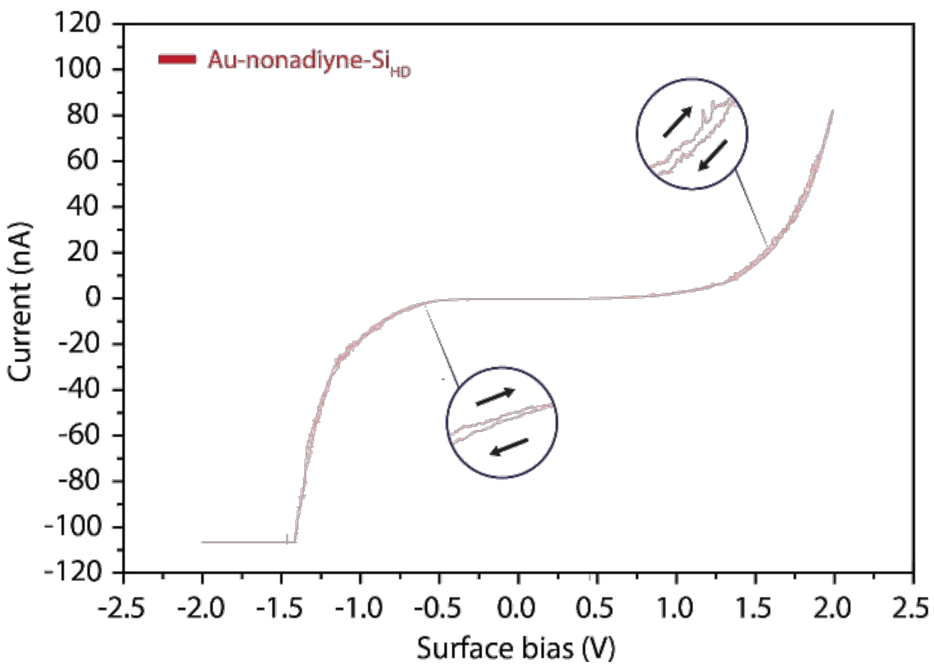

Supplementary Figure 3 **| IV characteristic of Au-(1,8-nonadiyne)-Si$_{LD}$ and Au-gap-Si junctions.** Characteristic I(V) curves showing both forward and backward directions of the voltage bias scan. No apparent hysteresis was observed at the voltage scan rate used (~16 V/s).

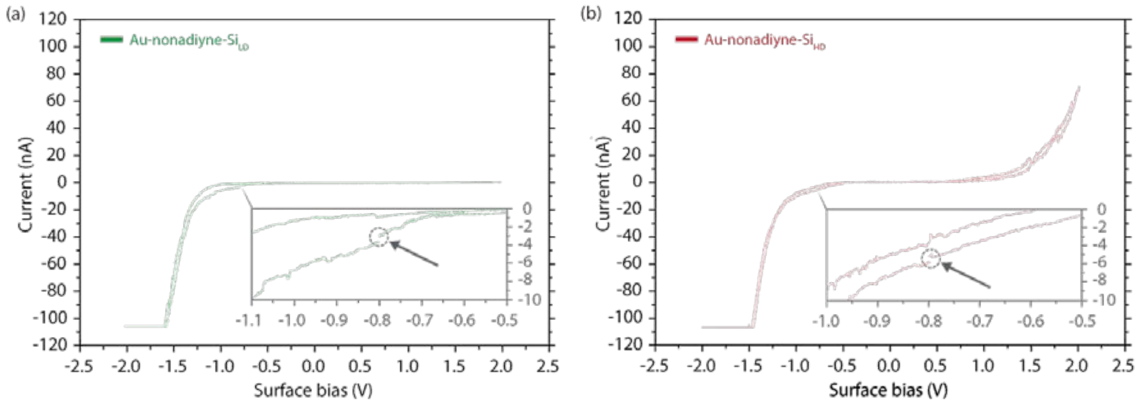

Supplementary Figure 4 **| IV curves showing the starting and ending bias.** The zoomed-in insets at -0.8 V show the trace starting and ending for (a) Au-nonadiyne-Si$_{LD}$ and (b) Au-nonadiyne-Si$_{HD}$.

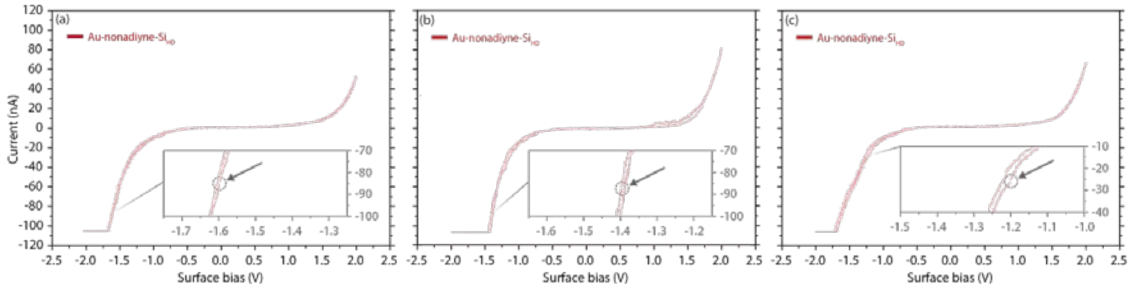

***Supplementary Figure 5* | IV curves at different starting set-bias.** The zoomed-in insets details the trace starting and ending points at -1.6 V (a), -1.4 V(b) and -1.2 V(c). The different bias starting points does not affect the behaviour of IVs.

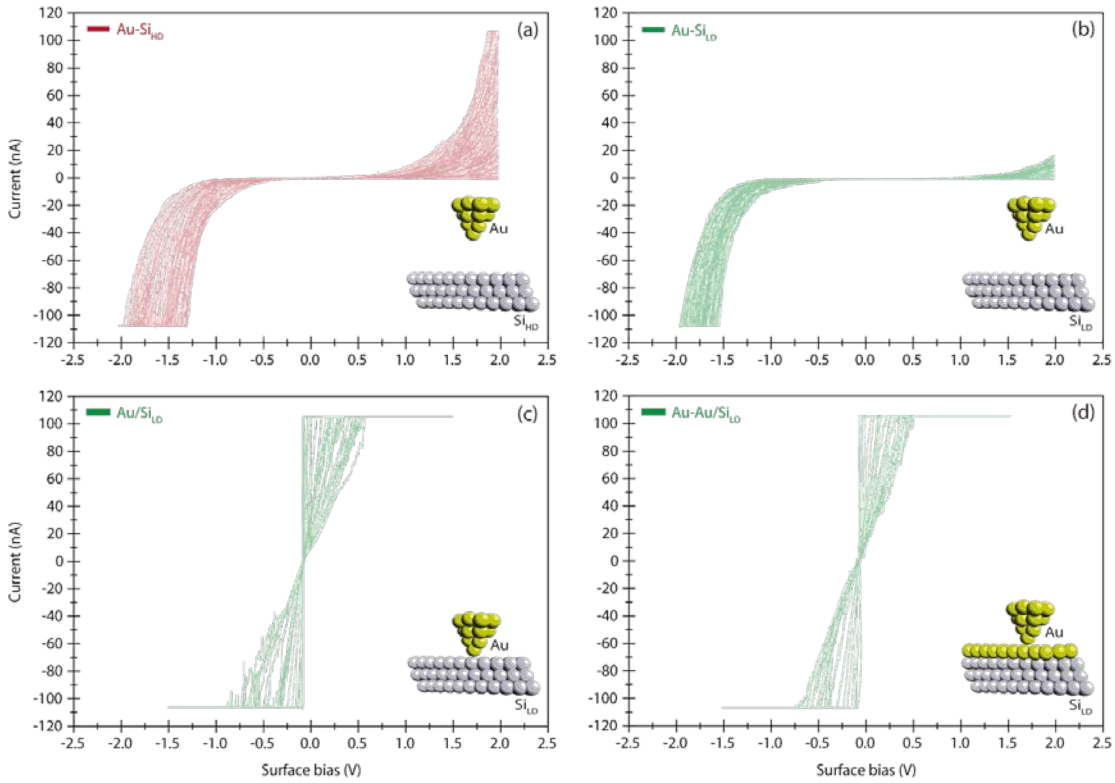

***Supplementary Figure 6* | IV characteristic of the Au-gap-Si junctions.** I(V)s characteristics of (a) an Au-Si_{HD} tunnelling gap (35 curves), (b) an Au-Si_{LD} tunnelling gap (35 curves), (c) and (d) Au/Si_{LD} and Au-Au/Si_{LD} respectively, both in direct physical contact showing an Ohmic behaviour. Both non-rectifying and rectifying behaviours can be observed in both (a) and (b) gap junctions with a large dispersion in the RR values, demonstrating the poor reproducibility of an open tunnelling gap under ambient conditions.

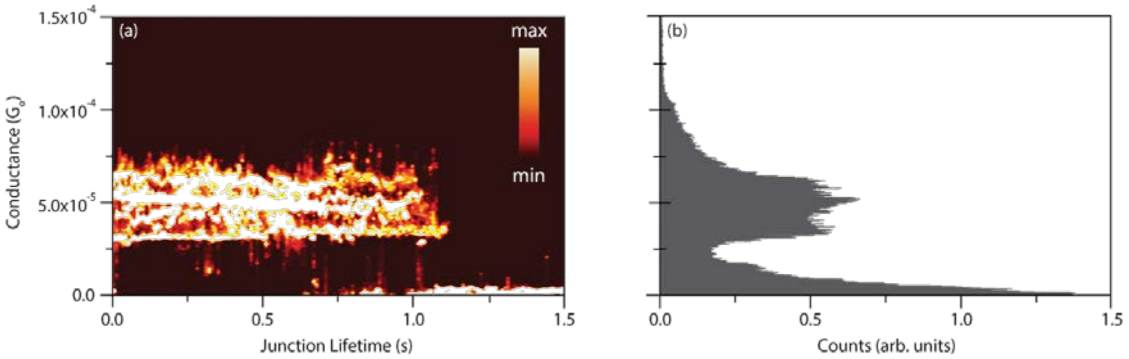

Supplementary Figure 7 **| Blinking experiments of Au-(1,8-nonadiyne)-Si$_{HD}$.** Comparison between the 2D blinking map (a) and the 1D histogram (b) of the same accumulated blinking Au-(1,8-nonadiyne)-Si$_{HD}$ captures. The applied surface-voltage bias and initial setpoint currents were -0.8 V and 1 nA, respectively.

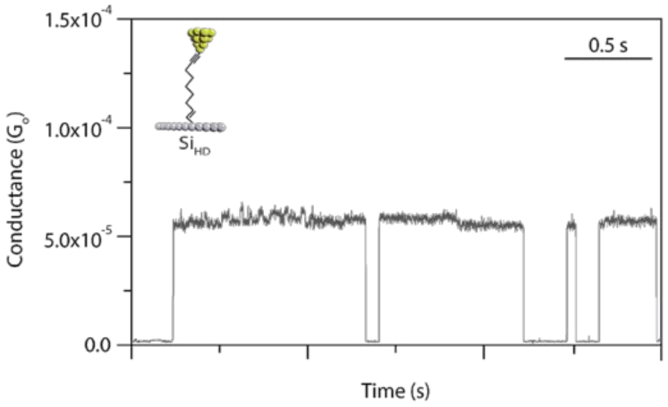

Supplementary Figure 8 **| Example of a high-frequency blinking of Au-(1,8-nonadiyne)-Si$_{HD}$.** We speculate that the non-homogenous and sudden increase in the frequency of the junction in the silicon case is attributed to the rigidity of the carbon-silicon bond which enables the same single molecule to form the Au-(1,8-nonadiyne)-Si$_{HD}$ junction multiple times in short period of time (breaking from the weak gold electrode and then reforming again) which lead to a sudden increase in the blinking frequency. The applied surface-voltage bias and initial set-point currents were -0.8 V and 1 nA, respectively and the current frame of the capture corresponds to 3 seconds.

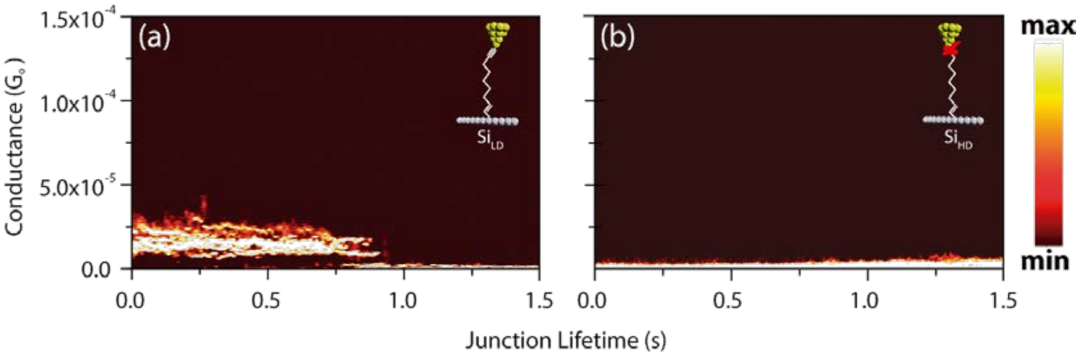

***Supplementary Figure 9* | 2D Blinking maps of Au-(1,8-nonadiyne)-Si_{LD} versus Au-(1-nonyne)-Si_{LD} junctions.** (a) 2D blinking maps obtained from hundreds of blinking events due to the formation of 1,8-nonadiyne single-molecule junctions between an Au tip and the low doped n-type silicon electrodes. (b) 2D blinking maps obtained from blinking experiments with 1-nonyne molecules (which possess no alkyne moieties at the distal end) showing no evidence of blinking events. Counts (color legend) have been normalized versus the total amount with 100% representing the maximum normalizing value. All used blinking traces were tunnelling-background subtracted and set to a common time origin.

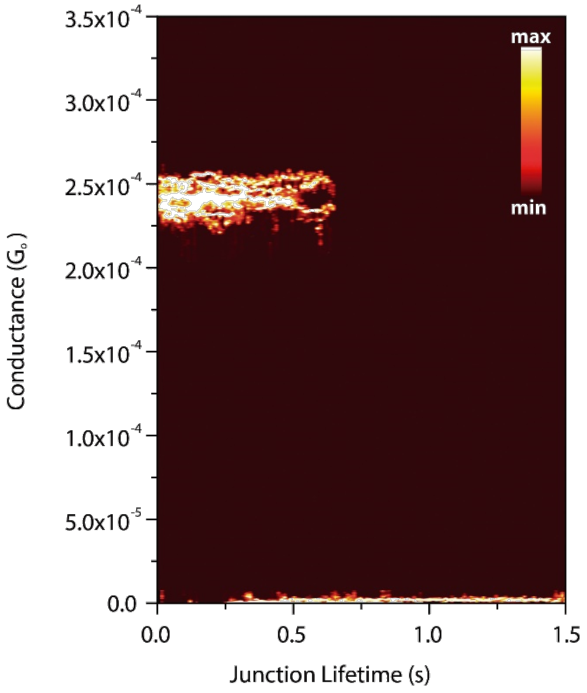

***Supplementary Figure 10* | 2D Blinking maps of Au-nonadiyne-Au junctions.** Traces obtained by the accumulation of hundreds of blinking events Au-1,8-nonadiyne-Au. Counts (color legend) have been normalized versus the total amount using 100% as the maximum normalizing value. All used blinking traces were set to a common time origin and tunneling background subtraction. See *Supplementary Note 2* for detailed information.

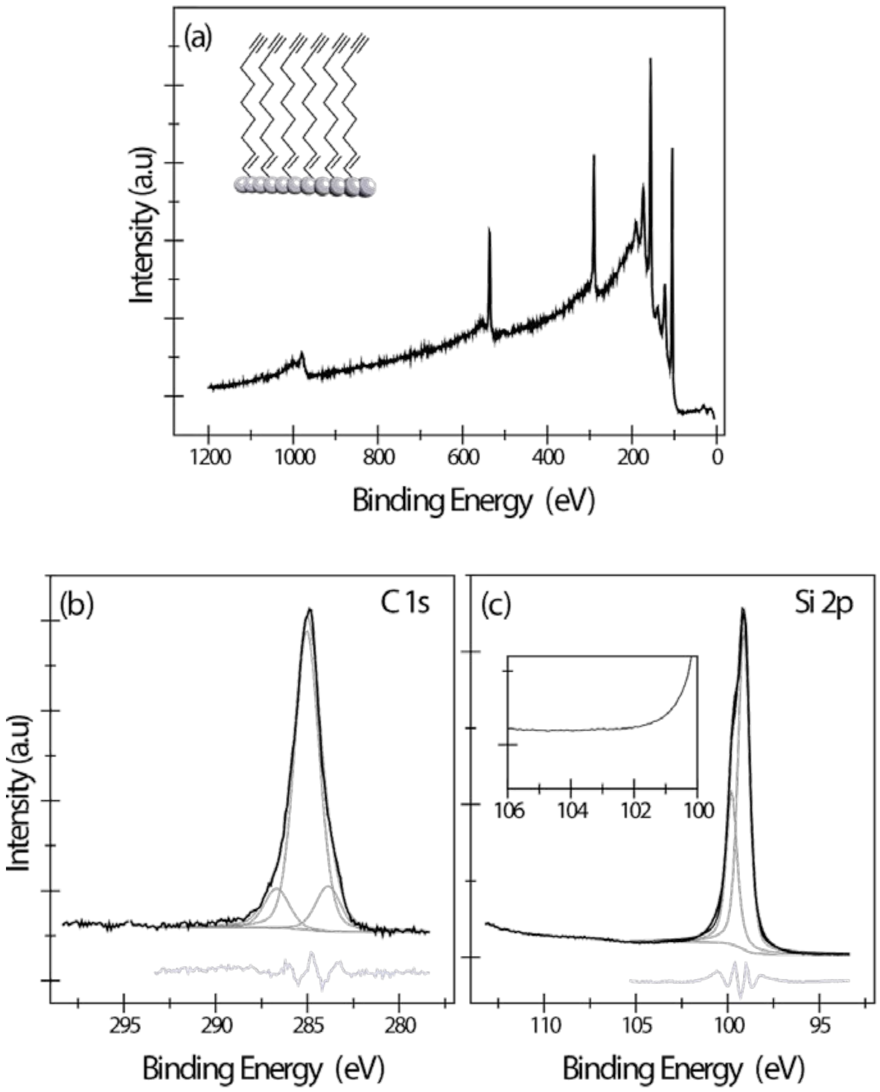

***Supplementary Figure 11* | XPS Spectrum of 1,8-nonadiyne monolayers.** (a) XPS spectra of monolayers assembled from the 1,8-nonadiyne on a hydrogen-terminated low doped n-type Si(111) sample. (b) Narrow scan of the C 1s region. The signal was fit with three components at 283.9, 285.0, and 286.7 eV comprising respectively of 1.2/10, 7.8/10, and 1.0/10 of the total C 1s signal. (c) High-resolution scan for the Si 2p region comprising two spin–orbit-split components. Silicon oxide-related emission was not observed; absent from the spectra (i.e., below spectrometer detection limit of ca. <0.05 SiOx monolayers equivalents) is the 102–104 eV signal associated with SiOx (see Panel c inset).

LUMO	HOMO
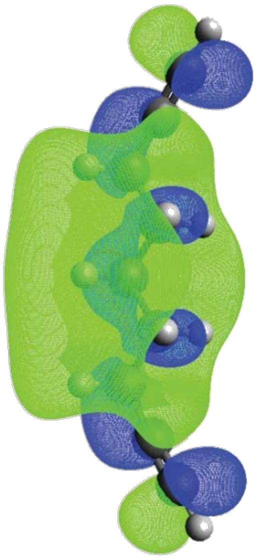	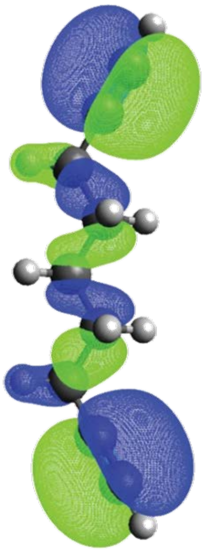
0.76 eV	-7.31 eV
**Band gap energy = 8.07 eV**	

***Supplementary Figure 12* | Calculations results for the 1,8-nonadiyne HOMO-LUMO gap.** Calculated energy values (in eV) of the represented HOMO and LUMO frontiers orbitals and the optimized geometries merged with the Isosurface plots (Isovalue = 0.02) of the nonadiyne molecule. The calculated frontier orbitals for the nonadiyne molecule were obtained with Density Functional (DFT) methods employing B3LYP functionals and using 6-311G(d) as basis set using the software package Gaussian 09.[1–4] All isosurface plots were obtained using Avogadro visualizing sofware.[5]

	Au-nonadiyne-Si_{LD}	Au-nonadiyne-Si_{HD}	Au-Si_{HD}	Au-Si_{LD}
C_V	0.74	1.39	8.40	5.94

***Supplementary Table 1* | Coefficient of variation study of IV curves of Si-Au junctions.** Coefficient of variation (C_V) of the 35 IV curves for Au-nonadiyne-Si_{LD} Au-nonadiyne-Si_{HD}, Au-Si_{HD} and Au-Si_{LD}. C_V was calculated using the following expression $C_V=\frac{\sigma}{\mu}$, where σ is the *standard deviation* and σ is the *mean*.

***Supplementary Note 1* |** *Supplementary Figure 1* shows current captures corresponding to blinking events representing the spontaneous formation of single-molecule junctions. The blinking experiments are performed under different applied surface-bias. As a consequence, there exist a variation in the observed current directly proportional to the applied surface-bias.

***Supplementary Note 2* |** *Supplementary Figure 10* shows a 2D blinking map corresponding to the formation of single-molecule junctions of 1,8-nonadiyne formed between two Au electrodes. The lifetime of Au-nonadiyne-Au junctions is 30% shorter than that of Au-nonadiyne-Si junctions due the weaker Au-C bond of the former.

Supplementary Methods

Characterization of the 1,8-nonadiyne monolayers using XPS. High-resolution Si 2p XPS spectra acquired on the acetylene-terminated **S-1** sample are shown in Fig. S4.1 and in agreement with previous reports.[6–8] As shown in Fig. S4.1c, the two spin–orbit-split refined contributions to the Si 2p doublet are located at 99.1 and 99.8 eV and associated to the $Si^{(0)}$ oxidation state.[9] Shifts toward higher binding energies for the Si 2p region are generally due to the presence of $Si^{(1)}$–$Si^{(4)}$ oxides and are well-documented.[10–12] For the 1,8-nonadiyne modified electrodes the silicon oxide content (102–104 eV)[13] was below the spectrometer detection limit, hence supporting the high quality of the monolayer. The high-resolution C 1s signals (Fig. S4.1b) were deconvoluted and fitted to three functions: i) a major contribution centered at 285.0 eV (78%, 1.6 eV fwhm) from aliphatic carbon-bonded carbon (C−C)[14,15] of the adsorbate peak, ii) a low binding energy contribution at 283.9 eV (12%, 1.6 eV fwhm) assigned to silicon-bound olefinic carbons (silylated olefin, Si–C̲=C)[16–19] and iii) an high binding energy signal at 286.7 eV (10%, 1.6 eV fwhm) associated to sp-hybridized carbon-bound carbon (–C≡C– groups).[18]

Technical details of the STM junction experiments. Details about the STM-break junction technique are published elsewhere.[20–22] All the conductance measurements were carried out with a mechanically and electronically isolated PicoSPM I microscope head controlled by a Picoscan-2500 electronics (all from Agilent, USA) and using a homemade PTFE STM cell. Data captures were acquired using a NI-DAQmx/BNC-2110 National Instruments, USA (LabVIEW data acquisition System) and analysed with LabVIEW code. The “blinking” modality of the STM break-junction technique has been published elsewhere.[23] The experiments detect the formation of molecular bridges between an Au STM tip and Si substrate electrodes while they are fixed at a specific electrode-electrode distance by imposing an initial setpoint tunnelling current. Once the tunnelling current is reached, the feedback current loop is turned off and the tunnelling current is monitored. Current jumps or “blinking” events appear due to the spontaneous formation of molecular bridges between the STM tip and substrate electrodes. The blinking data is plotted as 2D conductance maps by the accumulation of hundreds of these individual “blinking” events. All the “blinking” traces were accumulated to build the 2D maps without any selection. This accumulation process makes some conductance regions of the 2D map more prominent (more counts) above the tunnelling background as a consequence of the accumulated “blinking” traces. This provides an averaged value of the single-molecule conductance. Single molecule conductance (G) was determined using the expression $G=I_{step}/U_{BIAS}$, where I is the current and U is the voltage difference between the two junction electrodes. The 2D maps were built using an automatic selection procedure driven by a code written in LabVIEW. The 2D blinking maps were compiled by applying the same automated selection criteria to each set of captured data. The Current-voltage (I(V)) curves are obtained by the application of a “2-vertex voltage ramp” during the “blinking” event.[24] The “2-vertex voltage ramp” is applied and recorded by a code written in LabVIEW. The pulling procedure is based on the application of a “voltage ramp” to the piezoelectric translator that leads to the retraction of the tip, increasing the distance between both electrodes.[23] Pulling curves collected over the “blinking events” showed a plateau due to a significant mechanical resistance of the molecular junction between both electrodes. When pulling curves are collected over the tunnelling background, clean exponential decays are observed.

Supplementary References

1. Koch, W. & Holthausen, M. C. *A Chemist's Guide to Density Functional Theory*. *Neural Networks* **3,** (Wiley-VCH Verlag GmbH, 2001).

2. James B.Foresman, Ae. F. Exploring Chemistry With Electronic Structure Methods. *Gaussian,Inc Pittsburgh, PA* 335 (1995). doi:10.1002/adma.200400767

3. Savin, A. *Recent Developments and Applications of Modern Density Functional Theory*. *Theoretical and Computational Chemistry* **4,** (1996).

4. Frisch, M. J. *et al.* Gaussian 09, Revision A.02. *Gaussian Inc Wallingford CT* **34,** Wallingford CT (2009).

5. Hanwell, M. D. *et al.* Avogadro: An advanced semantic chemical editor, visualization, and analysis platform. *J. Cheminform.* **4,** 17 (2012).

6. Ciampi, S. *et al.* Functionalization of Acetylene-Terminated Monolayers on Si(100) Surfaces: A Click Chemistry Approach. *Langmuir* **23,** 9320–9329 (2007).

7. Michaels, P. *et al.* A robust DNA interface on a silicon electrode. *Chem. Commun. (Camb).* **50,** 7878–80 (2014).

8. Choudhury, M. H. *et al.* Connecting electrodes with light: one wire, many electrodes. *Chem. Sci.* **6,** 6769–6776 (2015).

9. Cordier, S. *et al.* Covalent Anchoring of Re 6 Se i 8 Cluster Cores Monolayers on Modified n- and p-Type Si(111) Surfaces: Effect of Coverage on Electronic Properties. *J. Phys. Chem. C* **114,** 18622–18633 (2010).

10. Cerofolini, G. F., Galati, C., Reina, S. & Renna, L. Grafting of 1-alkynes to hydrogen-terminated (100)silicon surfaces. *Appl. Phys. A* **80,** 161–166 (2005).

11. Himpsel, F. J., McFeely, F. R., Taleb-Ibrahimi, A., Yarmoff, J. A. & Hollinger, G. Microscopic structure of the SiO2/Si interface. *Phys. Rev. B* **38,** 6084–6096 (1988).

12. Ciampi, S., Guan, B., Darwish, N., Reece, P. J. & Gooding, J. J. Redox-Active Monolayers in Mesoporous Silicon. *J. Phys. Chem. C* **116,** 16080–16088 (2012).

13. Sieval, A. B., Linke, R., Zuilhof, H. & Sudhölter, E. J. R. High-Quality Alkyl Monolayers on Silicon Surfaces. *Adv. Mater.* **12,** 1457–1460 (2000).

14. Cerofolini, G. F. *et al.* Hydrosilation of 1-alkyne at nearly flat, terraced, homogeneously hydrogen-terminated silicon (100) surfaces. *Surf. Interface Anal.* **37,** 71–76 (2005).

15. Lehner, A., Steinhoff, G., Brandt, M. S., Eickhoff, M. & Stutzmann, M. Hydrosilylation of crystalline silicon (111) and hydrogenated amorphous silicon surfaces: A comparative x-ray photoelectron spectroscopy study. *J. Appl. Phys.* **94,** 2289 (2003).

16. Wallart, X., Henry de Villeneuve, C. & Allongue, P. Truly quantitative XPS characterization of organic monolayers on silicon: study of alkyl and alkoxy monolayers on H-Si(111). *J. Am. Chem. Soc.* **127,** 7871–8 (2005).

17. van Buuren, T., Tiedje, T., Dahn, J. R. & Way, B. M. Photoelectron spectroscopy measurements of the band gap in porous silicon. *Appl. Phys. Lett.* **63,** 2911 (1993).

18. Rijksen, B. *et al.* Hexadecadienyl monolayers on hydrogen-terminated Si(111): faster monolayer formation and improved surface coverage using the enyne moiety. *Langmuir* **28,** 6577–88 (2012).

19. Liu, H. & Hamers, R. J. An X-ray photoelectron spectroscopy study of the bonding of unsaturated organic molecules to the Si(001) surface. *Surf. Sci.* **416,** 354–362 (1998).

20. Xu, B. & Tao, N. J. Measurement of single-molecule resistance by repeated formation of molecular junctions. *Science* **301,** 1221–3 (2003).

21. Haiss, W. *et al.* Measurement of single molecule conductivity using the spontaneous formation of molecular wires. *Phys. Chem. Chem. Phys.* **6,** 4330–4337 (2004).

22. Haiss, W. *et al.* Precision control of single-molecule electrical junctions. *Nat. Mater.* **5,** 995–1002 (2006).

23. Aragonès, A. C. *et al.* Electrostatic catalysis of a Diels–Alder reaction. *Nature* **531,** 88–91 (2016).

24. Díez-Pérez, I. *et al.* Rectification and stability of a single molecular diode with controlled orientation. *Nat. Chem.* **1,** 635–641 (2009).
